# Epidemiology and Clinical Presentations of Respiratory Syncytial Virus Subgroups A and B Detected with Multiplex Real-Time PCR

**DOI:** 10.1371/journal.pone.0165108

**Published:** 2016-10-20

**Authors:** Wenkuan Liu, Dehui Chen, Weiping Tan, Duo Xu, Shuyan Qiu, Zhiqi Zeng, Xiao Li, Rong Zhou

**Affiliations:** 1 State Key Laboratory of Respiratory Diseases, The First Affiliated Hospital of Guangzhou Medical University, Guangzhou Medical University, Guangzhou, Guangdong, China; 2 Sun Yat-Sen Memorial Hospital, Sun Yat-Sen University, Guangzhou, Guangdong, China; National Heart and Lung Institute, UNITED KINGDOM

## Abstract

Respiratory syncytial virus (RSV) is one of the most important pathogenic infections of children and requires in-depth research worldwide, and especially in developing countries. We used a novel multiplex real-time PCR to test 5483 patients (≤ 14 years old) hospitalized with respiratory illness in Guangzhou, China, over a 3-year period. Of these patients, 729 were positive for RSV-A (51.2%, 373/729) or RSV-B (48.8%, 356/729), but none was infected with both viruses. Two seasonal peaks in total RSV were detected at the changes from winter to spring and from summer to autumn. RSV-B was dominant in 2013 and RSV-A in 2015, whereas RSV-A and RSV-B cocirculated in 2014. The clinical presentations of 645 RSV-positive patients were analyzed. Bronchiolitis, dyspnea, coryza, vomiting, poor appetite, and diarrhea occurred more frequently in RSV-A-positive than RSV-B-positive patients, whereas chill, headache, myalgia, debility, and rash etc. were more frequent in RSV-B-positive than RSV-A-positive patients, suggesting specific clinical characteristics for RSV-A and RSV-B. Coinfectons with other pathogens were common and diverse. Bronchiolitis, fever (≥ 38°C), and poor appetite were more frequent in patients with single RSV infections than in coinfected patients, suggesting the key pathogenic activity of RSV. Analysis of the relationships between the comparative viral load and clinical presentations showed significant differences in bronchiolitis, fever (≥ 38°C), and rash etc. among patients with different viral loads. This study provides a novel rapid method for detecting RSV subgroups, and provides new insights into the epidemiology and clinical implications of RSV.

## Introduction

Respiratory syncytial virus (RSV), of the family *Paramyxoviridae*, is a nonsegmented, negative-strand RNA virus that expresses 11 proteins [1]. RSV is amongst the most important pathogenic infections of childhood and is associated with significant morbidity and mortality, especially in developing countries [2]. The clinical manifestations of RSV infection range from mild upper-respiratory-tract illnesses (URTIs) or otitis media to severe and potentially life-threatening lower-respiratory-tract illnesses (LRTIs). The most common form of LRTI in RSV-infected infants is bronchiolitis, but pneumonia and croup are also seen [2–6]. There are two major antigenic subgroups of RSV, A and B, based on antigenic differences in their glycoproteins G and F [7–10]. The epidemiological features of RSV-A and RSV-B have differed in previous studies, and although strains of both subtypes often cocirculate, one subtypes generally predominates, depending on the region and climate [5, 6, 11, 12]. Therefore, the in-depth epidemiological characterization of RSV in specific areas is required worldwide, and especially in developing countries.

RSV is detected with direct or indirect immunofluorescence, enzyme-linked immunosorbent assay, viral culture, or increasingly, with reverse transcription–polymerase chain reaction (RT–PCR), especially real-time RT–PCR [6, 13]. Real-time PCR has many advantages in the detection of viruses, including its high specificity, high sensitivity, quantifiability, and simplicity of operation. It has been used for early diagnosis and the evaluation of drug efficacy and therapeutic effects, although the quantitative analysis of viruses in respiratory specimens is still impracticable. The results are usually simply positive or negative, although quantification is possible from Ct values or with plaque assays [14, 15]. The results from two independent respiratory specimens, such as throat swabs, nasopharyngeal swabs, or bronchial alveolar lavage fluid, can differ, even when both specimens are taken from the same individual at a same time, because they are dependent the sampling skill and features of the samples themselves. Thus, inconsistent results can be produced. However, comparative quantification PCR using a housekeeping gene, such as β-actin (*ACTB*) or glyceraldehyde 3-phosphate dehydrogenase (*GAPDH*), might be suitable for the analysis of these types of respiratory specimens.

In this study, we investigated the epidemiological features of RSV (subgroups A and B) in pediatric patients (≤ 14 years old) hospitalized with respiratory illness in Guangzhou, China, between January 2013 and December 2015, using a newly established multiplex real-time PCR, including the housekeeping gene, human *ACTB*.

## Materials and Methods

### Ethics Statement

The study was approved by The First Affiliated Hospital of Guangzhou Medical University Ethics Committee for research on human beings, and the next of kin, caretakers, or guardians on behalf of the minors/children gave signed informed consent for participation in the study.

### Respiratory sample collection

Throat swab samples from pediatric patients (≤ 14 years old) hospitalized with acute respiratory-tract illness (ARTI) were collected at two hospitals between January 2013 and December 2015 for routine screening for respiratory viruses, *Mycoplasma pneumoniae* (MP), and *Chlamydophila pneumoniae* (CP). The samples were collected with established clinical protocols [16]. The samples were refrigerated at 2–8°C in viral transport medium, transported on ice to the State Key Laboratory of Respiratory Diseases, and analyzed immediately or stored at −80°C before analysis as described in detail previously [17].

The patients’ clinical presentations were collected from their medical records using designed presentation cards, and were categorized retrospectively into the following six groups: URTI, LRTI, systemic influenza-like symptoms, gastrointestinal illness, neurological symptoms, and others. Patients with nasal obstruction, coryza, sneezing, coughing, pharyngeal discomfort, or hoarseness were categorized as having URTI. Patients with pneumonia, bronchiolitis, increasing lung markings, dyspnea, or an abnormal pulmonary breath sound were categorized as having LRTI. Patients with high fever (≥ 38°C), chills, dizziness, headache, myalgia, or debility were categorized as having systemic influenza-like symptoms. Patients with vomiting, poor appetite, or diarrhea were categorized as having a gastrointestinal illness. Patients with convulsions were categorized as having neurological symptoms. Patients with other symptoms, including but not limited to rash, were classified as “others”. Some patients were assigned to several clinical presentation groups. Increasing lung markings, pneumonia, and bronchiolitis were diagnosed with chest radiography. Abnormal pulmonary breath sounds included phlegmatic rales, wheezy rales, bubbling rales, and moist rales. Other clinical symptoms were identified with a normal medical examination and clinical descriptions.

### Multiplex real-time PCR to detect RSV-A, RSV-B, and human *ACTB*

RNA and DNA were extracted from the respiratory samples with the QIAamp Viral RNA Mini Kit and QIAamp DNA Mini Kit (Qiagen, Shanghai, China), respectively, in accordance with the manufacturer’s protocols. The primers and TaqMan probes designed to detect RSV subgroups A and B, by amplifying the RSV G gene, and to detect the mRNA of internal control, human *ACTB*, are listed in Table 1. Multiplex real-time RT–PCR was conducted using our optimized reaction buffer and cycling conditions. Each 25 μl reaction mixture contained 1× reaction buffer (50 mM Tris-Cl [pH 8.9], 75 mM KCl, 4 mM MgCl_2_, 10% glycerol), 0.6 mM deoxynucleoside triphosphates (dNTPs) (Promega, Beijing, China), 0.4 μM each primer (for RSV A, RSV B, and human *ACTB*) (BGI, Shenzhen, China), fluorescent probes for RSV A and RSV B (0.04 μM), fluorescent probe for human *ACTB* (0.06 μM) (TaKaRa, Dalian, China) (Table 1), 50 U of M-MLV reverse transcriptase (Promega, Beijing, China), 1 U of *Taq* DNA polymerase (Promega, Beijing, China), and 5 μl of template RNA. The cycling conditions were 48°C for 10 min, 94°C for 2 min, and then 40 cycles of 94°C for 10 s and 55°C for 35 s. The amplified nucleic acids were detected with the Applied Biosystems 7500 Real-Time PCR System (Life Technologies, Singapore).

**Table 1:** Primers and probes used in multiplex real-time PCR to detect RSV-A, RSV-B, and human *ACTB*.

Target ^a^	Primers and probes	Sequences ^b^
RSV-A		
	Forward	5’-ACTGCAATCAYACAAGATGCAACRA-3’
	Reverse	5’-CAGATTGRAGAAGCTGATTCCA-3’
	Probe	5’-FAM-CCAGATCAAGAACACAACCCCARCATACCT-BHQ1-3’
RSV-B		
	Forward	5’-ACTTACCTTACTCAAGTCTCACCAGAAA-3’
	Reverse	5’-TTGTRGCTGARTTTGTGTGGAT-3’
	Probe	5’-Texas red-TTAGCCCATCCMAACAAYCCACAACC-BHQ2-3’
Human beta actin		
	Forward	5’-TGGCACCCAGCACAATGA-3’
	Reverse	5’-GCCGATCCACACGGAGTACT-3’
	Probe	5’-JOE-ATCAAGATCATTGCTCCTCCTGAGCGC-BHQ1-3’

Note:

^a^ Conserved G gene specific for RSV-A and RSV-B, and human *ACTB* gene were used for detection.

^b^ TaqMan probes were used; Y represents C or T; R represents A or G; M represents A or C.

### Specificity of the multiplex real-time PCR in detecting RSV-A, RSV-B, and human *ACTB*

Each of 15 cultured RSV-A- or RSV-B-positive samples, stored at the State Key Laboratory of Respiratory Diseases and a panel of respiratory pathogens commonly found in the respiratory tract were used to determine the specificity of the multiplex real-time PCR. The nucleic acids were extracted from samples positive for RSV-A, RSV-B, influenza A virus (InfA), influenza B virus (InfB), four types of parainfluenza (PIV1–4), adenovirus (ADV), enterovirus (EV), human metapneumovirus (HMPV), four strains of human coronavirus (HCoV-229E, OC43, NL63, and HKU1), rhinovirus (RV), human bocavirus (HBoV), MP, CP, *Streptococcus pneumonia*, *Staphylococcus aureus*, and *Haemophilus influenza*, and tested with the multiplex real-time PCR.

### Sensitivity of multiplex real-time PCR in detecting RSV-A, RSV-B, and human *ACTB*

RNA transcripts of RSV-A, RSV-B, and human *ACTB* were used as standards to determine the assay sensitivity. Primers for RSV-A, RSV-B, and *ACTB* containing the T7 promoter sequence were used to amplify the target DNAs with the PrimeScript One Step RT–PCR Kit ver. 2 (TaKaRa, Dalian, China): RSV-A forward primer: 5′-TAATACGACTCACTATAACTGCAATCAYACAAGATGCAACRA-3′; RSV-A reverse primer: 5′-CAGATTGRAGAAGCTGATTCCA-3′; RSV-B forward primer: 5′-TAATACGACTCACTATAACTTACCTTACTCAAGTCTCACCAGAAA-3′; RSV-B reverse primer: 5′-TTGTRGCTGARTTTGTGTGGAT-3′; human *ACTB* forward primer: 5′-TAATACGACTCACTATAGGCATCCACGAAACTACCTT-3′; and human *ACTB* reverse primer: 5′-GCCGGACTCGTCATACTCCT-3′. The target DNAs were purified with a TIANprep DNA gel extraction kit (Tiangen, Beijing, China), and the RNAs were transcribed *in vitro* with a TranscriptAid T7 High Yield Transcription Kit (Thermo Scientific, Lithuania). The purity and concentrations of the RNA transcripts were established with a NanoDrop spectrophotometer (Thermo Scientific, NanoDrop Technologies, USA). The sensitivity and standard curves for the multiplex real-time PCR were established for RSV-A, RSV-B, and human *ACTB* by testing the transcripts of known concentrations serially diluted in diethyl-pyrocarbonate-treated water.

### Common respiratory pathogens detected in RSV-positive patients

RSV-positive samples were tested simultaneously for the following 17 respiratory pathogens: InfA, InfB, PIV1–4, ADV, EV, HMPV, HCoV-229E, HCoV-OC43, HCoV-NL63, HCoV-HKU1, RV, HBoV, MP and CP. The testing procedure, using kits from Guangzhou HuYanSuo Medical Technology Co., Ltd, has been described in a previous report [17].

### Comparative quantitation of RSV-A and RSV-B infections in throat swabs

Standard curves for the multiplex real-time PCR were constructed with RNA transcripts. The viral loads and the expression of human *ACTB* mRNA were calculated from the corresponding standard curves. The comparative viral loads (CVLs) of RSV-A and RSV-B were calculated by dividing the viral load by the amount of human *ACTB* transcripts in the same sample. CVL was used as the comparative quantity in the analysis.

### Statistical analysis

As described in detail previously [17], all statistical analyses were performed with the SPSS statistical software (version 19.0; SPSS Inc., Chicago, IL, USA). To compare categorical data, the χ^2^ test and Fisher’s exact test were used, as appropriate. All tests were two-tailed, and p < 0.05 was considered statistically significant.

## Results

### Specificity and sensitivity of multiplex real-time PCR in detecting RSV-A, RSV-B, and human *ACTB*

A panel of samples positive for RSV-A, RSV-B, and other common respiratory pathogens were tested with the multiplex real-time PCR, and no nonspecific reactions were observed. Serial dilutions of the target RNA transcripts were tested with the multiplex real-time PCR. The fluorescent signals observed at various dilutions of the two viral subgroups and human *ACTB* transcripts were used to calculate the minimal amounts of detectable RSV-A RNA (20 copies), RSV-B RNA (40 copies), and *ACTB* mRNA (60 copies) in 5 μl of RNA solution, and good linear relationships were found from 50 to 5 × 10^10^ copies of RSV-A (r^2^ = 0.9973), 500 to 5 × 10^10^ copies of RSV-B (r^2^ = 0.9989), and 500 to 5 × 10^10^ copies of *ACTB* mRNA (r^2^ = 0.9984) in 5 μl of RNA solution. The slopes of the standard curves were −3.30 for RSV-A, −3.35 for RSV-B, and −3.31 for human *ACTB* mRNA.

### Distributions of RSV-A and RSV-B in pediatric patients

In total, 5483 pediatric patients (≤14 years old) hospitalized with ARTI between January 2013 and December 2015were enrolled in this study. The male-to-female ratio was 1.74 (3481:2002) and the median age was 2.00 years (interquartile range, 0.75–4.00). Of the 5483 patients, 729 (13.3%) were positive for RSV-A or RSV-B, but no patient was coinfected with RSV-A and RSV-B. The male-to-female ratio was 2.41:1 (515:214) in the RSV-positive patients and 1.66:1 (2966:1788) in the RSV-negative patients (p < 0.001). The median age of the RSV-positive patients was 0.83 years (interquartile range, 0.42–2.00). Among these 729 patients, 373 (51.2%) were positive for RSV-A and 356 (48.8%) were positive for RSV-B. The male-to-female ratios were 2.33 (261:112) for RSV-A-positive patients and 2.49 (254:102) for RSV-B-positive patients. The median ages of the RSV-A- and RSV-B-positive patients were 0.83 years (interquartile range, 0.42–1.96) and 0.92 years (interquartile range, 0.42–2.00), respectively.

In this study, we tested the RSV-positive patients for another 17 common respiratory pathogens. Copathogens were common, and were found in 128 of the 729 (17.6%) RSV-positive patients: in 56 (15.0%, 56/128) RSV-A-positive patients and 72 (22.2%, 72/128) RSV-B positive patients (p = 0.065). Fifteen of these 17 pathogens (88.2%) were detected, and the most frequent copathogens were RV (26.6%, 34/128), InfA (21.1%, 27/128), EV (14.8%, 19/128), MP (11.7%, 15/128), ADV (10.9%, 14/128), HBoV (9.4%, 12/128), and HCoV-OC43 (8%, 6.3/128), followed by HMPV, InfB, PIV3, CP, PIV1, PIV4, HCoV-HKU1, and HCoV-229E at rates lower than 5%. PIV2 and HCoV-NL63 were not found in these patients (Table 2). MP was detected in 18.1% (13/72) of RSV-B-positive patients and 3.6% (2/56) of RSV-A-positive patients (p = 0.003) (Table 2). However, there were no significant differences in the detection rates for the other 16 copathogens in the RSV-A- or RSV-B-positive patients or the positive rates were too small to analyze (Table 2).

**Table 2:** Distribution of copathogens in RSV-positive patients.

Co-pathogen^a^	Total(n = 128)	Co-pathogen with RSV-A (n = 56)	Co-pathogen with RSV-B (n = 72)	p value^b^
RV	34(26.6)	14(25)	20(27.8)	0.233
infA	27(21.1)	11(19.6)	16(22.2)	0.269
EV	19(14.8)	10(17.9)	9(12.5)	0.897
MP	15(11.7)	2(3.6)	13(18.1)	**0.003**
ADV	14(10.9)	9(16.1)	5(6.9)	0.321
HBoV	12(9.4)	7(12.5)	5(6.9)	0.616
HCoV-OC43	8(6.3)	4(7.1)	4(5.6)	0.947
HMPV	6(4.7)	4(7.1)	2(2.8)	0.446
infB	3(2.3)	0(0)	3(4.2)	-^c^
PIV3	3(2.3)	2(3.6)	1(1.4)	-
CP	3(2.3)	0(0)	3(4.2)	-
PIV1	2(1.6)	0(0)	2(2.8)	-
PIV4	2(1.6)	0(0)	2(2.8)	-
HCoV-HKU1	2(1.6)	0(0)	2(2.8)	-
HCoV-229E	1(0.8)	0(0)	1(1.4)	-
PIV2	0(0)	0(0)	0(0)	-
HCoV-NL63	0(0)	0(0)	0(0)	-

Note: Data are number (%) in each group, except where specifically stated.

^a^ RSV-positive patients were tested for 17 common respiratory pathogens.

^b^ Two-tailed χ^2^ test, comparing the copathogen distribution between RSV-A-positive and RSV-B-positive patients.

^c^ Not done because the number of positive samples obtained was small.

**Fig 1:**
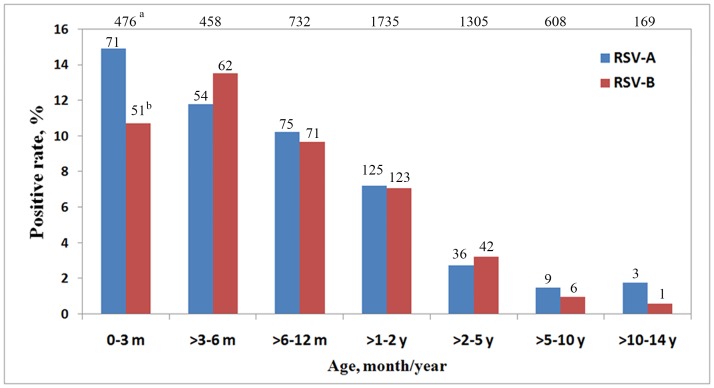
Age distributions of patients with RSV-A and RSV-B. ^a^Total number of patients in each age group; ^b^The number of RSV-A or RSV-B positive patients in each age group; m: month(s); y: year(s).

In this study, 710 of the 729 (97.4%) RSV-positive patients were children under 5 years old (p < 0.001), and 632 of the 729 (86.7%; p < 0.001) patients were under 2 years old. When the patients were divided into seven groups by age, the detection rate peaks for RSV-A and RSV-B occurred in patients aged 0–3 months (14.9%, 71/476) and >3–6 months (14.9%, 62/458), respectively, and the frequencies then declined with age (Fig 1).

**Fig 2:**
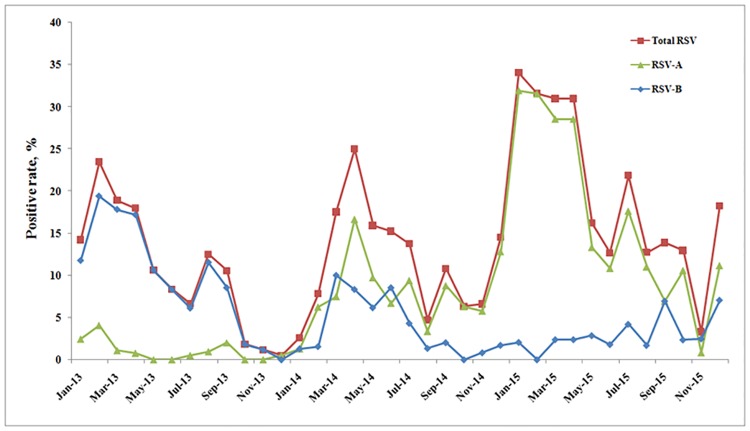
Seasonal distribution of RSV subgroups in pediatric patients hospitalized with acute respiratory-tract illness over a 3-year period. Note: Total RSV, sum of RSV-A and RSV-B; no patients were coinfected with RSV-A and RSV-B.

In general, the frequency of RSV infections peaked twice every year (Fig 2). Large peaks in the RSV detection rate occurred in February 2013 (23.5%, 23/98), April 2014 (25%, 66/264), and January 2015 (34.0%, 33/97). Small peaks in the RSV detection rates occurred in August 2013 (12.5%, 26/208), September 2014 (10.8%, 16/148), and July 2015 (21.8%, 26/119). RSV-B circulated predominantly in 2013 and RSV-A in 2015, whereas in 2014, RSV-B and RSV-A cocirculated (Fig 2).

### Distribution of clinical presentations in RSV-A- and RSV-B-positive patients

We analyzed the clinical presentations of 645 of the 729 (88.5%) RSV-positive patients. Patients with incomplete clinical data were excluded from this analysis. We compared the clinical characteristics of the RSV-A- and RSV-B-positive patients, the characteristics of single RSV infections and coinfections, and the characteristics of patients with different CVLs (Table 3).

**Table 3:** Clinical presentations in RSV-positive patients.

	Patients with RSV-A and RSV-B			Patients with single RSV-A and co-pathogens			Patients with different comparative viral load^d^				
Characteristics	RSV-A (n = 327)	RSV-B (n = 318)	p value^b^	Single RSV (n = 540)	Co-pathogens(n = 105)	p value^c^	<1 (n = 269)	1–8 (n = 228)	8–64 (116)	>64 (n = 32)	p value^e^
**URTI**											
Nasal obstruction	125(38.2)	107(33.6)	0.226	200(37)	32(30.5)	0.2	105(39)	82(36)	36(31)	10(31.3)	0.454
Coryza	164(50.2)	111(34.9)	**<0.001**	230(42.6)	45(42.9)	0.96	122(45.4)	100(43.9)	44(37.9)	9(28.1)	0.191
Sneeze	6(1.8)	5(1.6)	0.797	10(1.9)	1(1)	0.515	6(2.2)	4(1.8)	1(0.9)	0(0)	0.684
Cough	323(98.8)	307(96.5)	0.06	528(97.8)	102(97.1)	0.693	265(98.5)	219(96.1)	114(98.3)	32(100)	0.22
Expectoration	47(14.4)	53(16.7)	0.421	82(15.2)	18(17.1)	0.612	37(13.8)	39(17.1)	22(19)	2(6.3)	0.238
Pharyngeal discomfort	2(0.6)	1(0.3)	0.579	3(0.6)	0(0)	0.444	1(0.4)	2(0.9)	0(0)	0(0)	0.664
Hoarseness	4(1.2)	3(0.9)	0.732	6(1.1)	1(1)	0.886	3(1.1)	1(0.4)	3(2.6)	0(0)	0.229
**LRTI**											
Abnormal pulmonary breath sound ^a^	322(98.5)	310(97.5)	0.373	528(97.8)	104(99)	0.397	266(98.9)	224(98.2)	112(96.6)	30(93.8)	0.151
Dyspnoea	211(64.5)	181(56.9)	**0.048**	323(59.8)	69(65.7)	0.257	164(61)	137(60.1)	67(57.8)	24(75)	0.361
Increasing lung markings	40(12.2)	38(11.9)	0.912	64(11.9)	14(13.3)	0.67	38(14.1)	24(10.5)	13(11.2)	3(9.4)	0.598
Bronchiolitis	189(57.8)	125(39.3)	**<0.001**	274(50.7)	40(38.1)	**0.018**	148(55)	107(46.9)	50(43.1)	9(28.1)	**0.009**
Pneumonia	22(6.7)	14(4.4)	0.198	28(5.2)	8(7.6)	0.32	15(5.6)	14(6.1)	6(5.2)	1(3.1)	0.91
**Systemic influenza-like symptoms**											
Fever (≥38°C)	229(70)	230(72.3)	0.52	393(72.8)	66(62.9)	**0.04**	211(78.4)	155(68)	77(66.4)	16(50)	**0.001**
Chill	12(3.7)	27(8.5)	**0.01**	36(6.7)	3(2.9)	0.134	14(5.2)	16(7)	8(6.9)	1(3.1)	0.719
Dizziness	2(0.6)	4(1.3)	0.393	5(0.9)	1(1)	0.979	3(1.1)	1(0.4)	1(0.9)	1(3.1)	0.498
Headache	2(0.6)	12(3.8)	**0.006**	12(2.2)	2(1.9)	0.838	4(1.5)	5(2.2)	4(3.4)	1(3.1)	0.655
Myalgia	1(0.3)	12(3.8)	**0.002**	11(2)	2(1.9)	0.93	2(0.7)	6(2.6)	4(3.4)	1(3.1)	0.256
Debilitation	2(0.6)	12(3.8)	**0.006**	12(2.2)	2(1.9)	0.838	3(1.1)	6(2.6)	4(3.4)	1(3.1)	0.446
**Gastrointestinal illness**											
Vomiting	79(24.2)	45(14.2)	**0.001**	109(20.2)	15(14.3)	0.16	51(19)	48(21.1)	22(19)	3(9.4)	0.474
Poor appetite	121(37)	72(22.6)	**<0.001**	172(31.9)	21(20)	**0.015**	89(33.1)	63(27.6)	35(30.2)	6(18.8)	0.288
Diarrhoea	50(15.3)	26(8.2)	**0.005**	68(12.6)	8(7.6)	0.148	32(11.9)	26(11.4)	14(12.1)	4(12.5)	0.996
**Neurologic symptom**											
Convulsion	10(3.1)	8(2.5)	0.676	15(2.8)	3(2.9)	0.964	7(2.6)	7(3.1)	3(2.6)	1(3.1)	0.988
**Others**											
Rash etc	3(0.9)	31(9.7)	**<0.001**	22(4.1)	12(11.4)	**0.002**	9(3.3)	10(4.4)	11(9.5)	4(12.5)	**0.02**

Note: Data are number (%) in each group, except where specifically stated. Percentages sum to over 100% because some patients had more than one diagnosis.

^a^ Including phlegmatic rales, wheezing rales, bubbling rales, and moist rales.

^b^ Two-tailed χ^2^ test, comparing the distribution of each illness or diagnosis between the RSV-A-positive and RSV-B-positive patients.

^c^ Two-tailed χ^2^ test, comparing the distribution of each illness or diagnosis between patients singly infected with RSV and coinfected patients.

^d^ Comparative viral loads of RSV-A and RSV-B were calculated by dividing the viral load by the copies of human *ACTB* mRNA, which were calculated from standard curves, and used as comparative quantities for analysis.

^e^ Two-tailed χ^2^ test, comparing the distribution of each illness or diagnosis in patients with different comparative viral loads.

Significant differences were found between the RSV-A- and RSV-B-positive patients in the frequency of coryza (p < 0.001), dyspnea (p = 0.048), bronchiolitis (p < 0.001), chill (p = 0.01), headache (p = 0.006), myalgia (p = 0.002), debility (p = 0.006), vomiting (p = 0.001), poor appetite (p < 0.001), diarrhea (p = 0.005), and rash etc. (p < 0.001) (Table 3).

There were significant differences in the clinical characteristics of patients solely infected with RSV (RSV-A or RSV-B) and those coinfected, in the frequencies of bronchiolitis (p = 0.018), fever (≥ 38°C) (p = 0.04), poor appetite (p = 0.015), and rash etc. (p = 0.002) (Table 3).

In this study, CVL was divided into four levels: < 1, 1–8, 8–64, and > 64. Significant differences in the clinical characteristics of patients with different CVLs were found for bronchiolitis (p = 0.009), in which 55% of patients (148/269) were CVL < 1, 46.9% (107/228) were CVL = 1–8, 43.1% (50/116) were CVL = 8–64, and 28.1% (9/32) were CVL > 64; fever (≥ 38°C) (p = 0.001), in which 78.4% (211/269) were CVL < 1, 68% (155/228) were CVL = 1–8, 66.4% (77/116) were CVL = 8–64, and 50% (16/32) were CVL > 64; and rash etc. (p = 0.02), in which 3.3% (9/269) were CVL < 1, 4.4% (10/228) were CVL = 1–8, 9.5% (11/116) were CVL = 8–64, and 12.5% (4/32) were CVL > 64 (Table 3).

## Discussion

RSV is one of the most important pathogenic infections of children and is associated with significant morbidity and mortality [18–20]. RSV infection is a global epidemic, imposing a high public-health burden, and circulates with different patterns in different areas [21, 22]. Therefore, research into the epidemiological behavior of RSV, especially in developing countries, is essential. In this study, we analyzed the epidemiological characteristics of RSV-A and RSV-B infections in pediatric patients (≤ 14 years old) hospitalized with respiratory illness in Guangzhou, China over a 3-year period, using our newly established multiplex real-time PCR.

In this study, the multiplex real-time PCR amplified the G gene conserved in and specific to RSV-A and RSV-B and the human *ACTB* gene. The specificity and sensitivity of the method were analyzed. No nonspecific reactions were observed, and good linear relationships were observed for the three target genes. Parallel standard curves (with almost the same slopes) increased the quality of the CVL calculation.

In this 3-year study, 13.3% (729/5483) of patients were RSV positive, most were children under 2 years old (86.7%, 632/729) (p < 0.001), and more male patients than female patients were infected with RSV (p < 0.001) (Fig 1). This distribution is consistent with previous reports of RSV in both developed and developing countries [3, 23, 24]. The prevalences of RSV-A (51.2%, 373/729) and RSV-B (48.8%, 356/729) were similar in this study, and showed the same tendencies with age, insofar as most positive patients under 2 years old (Fig 1). The pattern in the seasonal distributions of RSV-A and RSV-B was found. RSV-B was the dominant subtype in 2013, RSV-A was dominant in 2015, and RSV-A and RSV-B cocirculated in 2014 (Fig 2). RSV is known to occur in well-defined recurrent epidemics during the cold season in temperate climates [2], whereas in tropical and subtropical areas, RSV infections are reported to peak more often in the wet season. However, locations close to the equator show less consistent patterns, some with almost continuous RSV activity and varying seasonal peaks [25]. In this study, two seasonal peaks of RSV occurred at the changes of seasons from winter to spring and from summer to autumn. This pattern is similar to the previous report [24].

Copathogens were common (17.6%, 128/729) and diverse (88.2%, 15/17) in the RSV-positive patients, as reported previously [2, 26–29], and the most frequently detected copathogens were RV, InfA, EV, MP, ADV, HBoV, and HCoV-OC43, which all occurred at rates > 5% (Table 2). When we compared the clinical presentations of patients with single RSV infections and those with coinfections, more bronchiolitis (p = 0.018), fever (≥ 38°C) (p = 0.04), and poor appetite (p = 0.015) were present in the singly infected patients than in the co-infected patients, suggesting the key pathogenic activity of RSV. On the contrary, more rash etc. (p = 0.002) was observed in the co-infected patients than in those with single RSV infections, indicating the nonmainstream activity of rash etc. in patients with RSV infection (Table 3). However, the knowledge of viral–viral interactions *in vivo* are poorly understood, and the clinical relevance of the infection of copathogens in these patients and their association with severe illness are unclear [26, 29].

RSV infection has a large spectrum of clinical manifestations, ranging from URTI to LRTI. In this study, we analyzed the clinical presentations of 645 RSV-positive patients (Table 3). The relationships between the clinical characteristics and the RSV subgroups (RSV-A, RSV-B) differed from previously reported results. Some studies found no differences in the symptoms of patients infected with the two RSV subtypes [30–32]. However, some reported that RSV-A was associated with a more-severe clinical disease [33–35], whereas in other studies, RSV-B infections were reported to cause more severe disease [36, 37]. In this study, the LRTIs bronchiolitis (p < 0.001) and dyspnea (p = 0.048), the URTI coryza (p < 0.001), and the gastrointestinal illnesses vomiting (p = 0.001), poor appetite (p < 0.001), and diarrhea (p = 0.005) were more frequently found in the RSV-A-positive patients than in the RSV-B-positive patients. However, the systemic influenza-like symptoms chill (p = 0.01), headache (p = 0.006), myalgia (p = 0.002), and debility (p = 0.006), and rash etc. (p < 0.001) were found more frequently in the RSV-B-positive patients than in the RSV-A-positive patients (Table 3). These data suggest specific clinical presentations for RSV-A and RSV-B infections, and provide clues for the diagnosis and clinical distinction of the different RSV subtypes.

Many factors affect the clinical manifestations of patients, including the infectious organism, environment, nutrition, and socioeconomic status. Viral infections are an important issue that attracts much research attention, and one focus of research is the relationship between the viral load and disease progression or the sensitivity of the disease. In the study of patients chronically infected with the hepatitis B virus, monitoring the homogeneous serum hepatitis B viral DNA levels with sensitive, absolutely quantitative real-time PCR assays is strongly recommended for the management of patients [38]. However, it is still challenging to quantify respiratory viruses in respiratory-tract specimens. Whether the RSV load correlates with disease severity remains controversial, because some previous analyses of hospitalized children have shown a significant association [15, 39–41], whereas others have not [42, 43]. The virus was quantified with quantitative RT–PCR, plaque assay, or both in these studies. However, the initial quality of the samples influences their subsequent analysis, and it is difficult to control how many valid cells can be obtained from the respiratory tract. To exclude the effects of sampling, a comparative quantitative analysis of viral load should be used for respiratory specimens to evaluate drug efficacy, therapeutic effects, etc.

In this study, we established a multiplex real-time PCR that included the housekeeping gene, human *ACTB*. The detection of human *ACTB* was used first to monitor the quality of sampling, and secondly, as a baseline for the calculation of viral load in the respiratory specimens. We thus made a limited attempt to analyze the association between CVL and clinical presentation. RSV (-A or -B) and human *ACTB* were quantified with a standard curve for each. The CVL was then calculated and used for the analysis. In this study, CVL was divided into four levels: < 1, 1–8, 8–64, and > 64. Significant differences were found in bronchiolitis (p = 0.009) and fever (≥ 38°C) (p = 0.001) according to the CVL, with the frequencies declining as the CVL increased (Table 3). The peak frequencies of bronchiolitis and fever occurred in low-CVL patients, which might attributable to the combined effects of the lung damage caused by the virus, the host immunopathological effect, the replication/clearance of the virus, the progression of the disease, etc. [44–46]. In the advanced stage of RSV infection, virus is gradually cleared, and immunopathological effect becomes the more important influence factor. While the disease progressions of the patients before hospitalization, like the onset of disease, were not recorded clearly, the shortcoming made it hard to analyze deeply, and more mechanism study must be done to uncover the inner relationship.

Significant differences were also seen in the frequency of rash etc. (p = 0.02), which increased as CVL increased (Table 3). However, other factors or pathogens might be responsible for this trend because rash occurred more often in patients with co-pathogen infections than in those with single RSV infections (p = 0.002) (Table 3).

In this work, a novel rapid method for detecting RSV subgroups was established, and the epidemiology and clinical manifestations of RSV infection in Guangzhou, China were analyzed. CVL was used as the comparative quantity in the analysis, while more studies, especially mechanism studies and longitudinal studies, should investigate the feasibility of new CVL evaluation methods, similar to the preliminary attempt made in this cross-sectional study.
